# Spontaneous Reperfusion after *In Situ* Thromboembolic Stroke in Mice

**DOI:** 10.1371/journal.pone.0050083

**Published:** 2012-11-16

**Authors:** Anne Durand, Fabien Chauveau, Tae-Hee Cho, Radu Bolbos, Jean-Baptiste Langlois, Laure Hermitte, Marlène Wiart, Yves Berthezène, Norbert Nighoghossian

**Affiliations:** 1 Université de Lyon, CREATIS, CNRS UMR5220, INSERM U1044, INSA-Lyon, Université Lyon 1, Hospices Civils de Lyon, Lyon, France; 2 CERMEP-Imagerie du Vivant, Animage, Lyon, France; Charité Universitaetsmedizin Berlin, Germany

## Abstract

Injection of thrombin into the middle cerebral artery (MCA) of mice has been proposed as a new model of thromboembolic stroke. The present study used sequential multiparametric Magnetic Resonance Imaging (MRI), including Magnetic Resonance Angiography (MRA), Diffusion-Weighted Imaging (DWI) and Perfusion-Weighted Imaging (PWI), to document MCA occlusion, PWI-DWI mismatch, and lesion development. In the first experiment, complete MCA occlusion and reproducible hypoperfusion were obtained in 85% of animals during the first hour after stroke onset. In the second experiment, 80% of animals showed partial to complete reperfusion during a three-hour follow-up. Spontaneous reperfusion thus contributed to the variability in ischemic volume in this model. The study confirmed the value of the model for evaluating new thrombolytic treatments, but calls for extended MRI follow-up at the acute stage in therapeutic studies.

## Introduction

A new rodent model of stroke was recently proposed by Orset *et al.* [1], in which thromboembolic middle cerebral artery occlusion (MCAo) was achieved by *in situ* microinjection of thrombin. Intravenous injection of tissue-type plasminogen activator induced reperfusion, mimicking clinical thrombolysis. Thus, this animal model appears very attractive for evaluating new thrombolytic agents or combined thrombolytic/neuroprotective strategies, as described in recent histopathologic studies [2], [3].

MRI plays a pivotal role in stroke management [4], and is increasingly used for treatment assessment in animal models [5], [6], [7], [8]. The present study sought to characterize acute development of ischemic infarct using MRI to assess the adequacy of the model.

## Materials and Methods

All procedures were conducted after approval (n°0237) by our institutional review board (*Comité Régional d’Ethique pour l’Expérimentation Animale* (CREEA Rhône-Alpes) of the CNRS), in compliance with European law and policies (EEC Council Directive 86/609, OJ L 358, 1, Dec. 12, 1987), and in accordance with the ARRIVE (Animal Research: Reporting In Vivo Experiments) guidelines (http://www.nc3rs.org/ARRIVE). Animals were housed in a colony room with a 12/12 h light/dark cycle.

### Animal Model

Twenty-six Swiss mice (Charles River, l’Arbresle, France), weighing 25–30 g, were studied. A catheter was inserted into the tail vein. To minimize suffering, buprenorphine (0.05 mg/kg s.c.) was administered shortly after induction of anesthesia with 2% isoflurane in ambient air. Body temperature was continuously maintained at 37.0±0.5°C. Surgery was performed as originally described [1]. Briefly, subtemporal craniectomy exposed the right MCA, and one microliter of murine α-thrombin (0.75 U, Sigma-Aldrich) was pneumatically injected into the lumen of the MCA. *In situ* clot formation was monitored visually. Animals were sutured and immediately placed in the MRI cradle.

### Magnetic Resonance Imaging

Mice were placed in a cradle equipped with a stereotaxic holder, an integrated heating system to maintain body temperature at 37±1°C, and a pressure probe to monitor respiration. MRI experiments were performed on a BioSpec-70/20 7T system (Bruker, Ettlingen, Germany) using a birdcage head-coil of 72 mm inner diameter for RF transmission and a 15 mm diameter surface coil for reception. A whole-brain imaging protocol (T2-weighted imaging (T2-WI), diffusion-weighted imaging (DWI) and perfusion-weighted imaging (PWI)) was adapted from a previous study [6], and performed during the first hour following stroke onset. Time of flight (TOF) MRA was performed using a 2D multislice gradient-echo sequence with matrix = 256×128, field of view (FOV) = 3.7×2.5 cm^2^, 80 non-contiguous slices (slice thickness 1 mm and interslice overlap 0.15 mm), echo time/repetition time (TE/TR) = 5/25 ms and 2 averages. T2-WI sequences were acquired using a RARE sequence with matrix = 256×256, FOV = 3×3 cm^2^, TE/TR = 75/3000 ms, and 2 averages. DWI was performed using an echo planar imaging spin-echo (EPI-SE) sequence with the same FOV, matrix 128×128, TE/TR = 25/2000 ms and 3 b-values of 0, 1,500, and 3,000 s/mm2. PWI was performed either i) with multislice dynamic susceptibility contrast-enhanced MRI (DSC-MRI, Experiment 1) or ii) with a single-slice pulsed arterial spin labeling method (PASL, Experiment 2). DSC-MRI consisted in an echo planar imaging gradient-echo (EPI-GE) sequence with the same FOV, matrix 96×96, zero-filled to 128×128, TE/TR = 4.84/100 ms, and 100 consecutive images per slice with a temporal resolution of 0.6 s, under an intravenous bolus of gadolinium (Dotarem, Guerbet, Aulnay-sous-Bois, France). PASL was performed using a flow-sensitive alternating inversion-recovery echo planar imaging (FAIR-EPI) sequence with matrix 96×48, FOV = 2×1 cm^2^, inversion recovery time (TIR) = 40 to 2,500, number of TIR values = 22, recovery time = 10,000 ms, TE/TR = 10/18,000 ms. T2-WI, DWI and DSC-PWI were performed using the same set of 7 non-contiguous slices (slice thickness 0.7 mm and interslice gap 0.3 mm), covering the entire MCA territory, while PASL was performed using the central slice only.

### Image Analysis

MRI analysis was performed by an investigator blind to surgery, using MIPAV software (Medical Image Processing, Analysis, and Visualization, NIH, Bethesda, MD, USA; http://mipav.cit.nih.gov/). Source images and maximum intensity projection for MRA were used to assess MCA occlusion. Apparent diffusion coefficient maps (ADC, in mm^2^/s) were calculated from native DWI by fitting MR signal curves to a mono-exponential model function on a pixel-by-pixel basis, using in-house software written in Matlab 2009 (MathWorks, Natick, MA, USA). Contrast-agent-induced signal intensity changes from DSC-MRI images were converted into changes in the transverse relaxation rate 1/T2* (i.e., ΔR2*) according to: ΔR2* = −1/TE.ln[S(t)/S0], where S0 is the pre-contrast MR signal intensity and S(t) the MR signal intensity at time t. Time-to-peak maps are commonly used in clinical settings to assess the hypoperfused area [9]; however, their low temporal resolution compared to bolus first-pass duration hampered their use in the present rodent study. Therefore, perfusion maps were generated with Matlab 2009 (MathWorks, Natick, MA, USA) using the peak of ΔR2*-time curves, or maximum peak concentration (MPC, in a. u.) [10]. PASL images were analyzed with the ASL_Perfusion_Processing macro from the Paravision 5.1 software platform (Bruker, Ettlingen, Germany), using a 7T blood T1 value of 2,200 ms [11]: cerebral blood flow (CBF, in ml/[min×100 g]) was derived from the non-selective and selective T1 maps according to CBF = λ.T1non-selective/T1blood.(1/T1selective-1/T1non-selective), where λ is the blood-brain partition coefficient: i.e., the ratio between water concentration per gram of brain tissue and per ml of blood, estimated at 90 ml/100 g [12]. Whole-brain tissue, ipsilateral and contralateral hemispheres were manually drawn on T2-weighted images and reported on computed maps. ADC maps were thresholded at the previously reported value of 0.53×10^−3^ mm^2^/s [13]. MPC and CBF maps were thresholded at the 10^th^ percentile value of perfusion in the contralateral hemisphere [14]. ADC, MPC and CBF defect volumes were calculated as the thresholded volume in the ipsilateral hemisphere minus the thresholded volume in the contralateral hemisphere. All lesion volumes were expressed as a percentage of the ipsilateral hemisphere volume.

### Study Design

#### Experiment 1: Acute characterization

Twenty mice underwent a single whole-brain imaging protocol (T2-WI, DWI and DSC-PWI with gadolinium injection) immediately after stroke onset. They were euthanized 24 hours after ischemia induction. Coronal brain sections (20 µm) from snap-frozen brain tissue were stained with cresyl violet. Seven sections taken at equally spaced 1 mm intervals, corresponding to MRI slices, were selected and digitized. Histological infarct volumes were manually delineated by an investigator blind to surgery and MRI analysis, and were expressed as a percentage of the ipsilateral hemisphere volume. Normalization to the entire ipsilateral hemisphere coped with tissue deformation of various sources (edema, fixation, histological processing) and enabled comparison between *in vivo* and *post mortem* data.

#### Experiment 2: Extended follow-up

Six mice underwent a 3-session MRI protocol to determine lesion evolution. The first session (T2-WI, DWI and PASL-PWI) was performed immediately after stroke onset and repeated after 3 hours. PASL-PWI was preferred to DSC-PWI so as not to repeat gadolinium injection. The third session (T2-WI) was performed after 24 hours, and animals were then sacrificed.

### Statistical Analysis

Data analysis was performed using SPSS software (Version 15.0, SPSS Inc., Chicago, IL, USA), with the statistical significance threshold set at P<0.05. All lesion volumes are reported as mean±standard deviation. Comparisons were performed with non-parametric Kruskal-Wallis tests. Mann–Whitney tests were used for *post hoc* paired comparisons. Correlations were determined by Spearman’s rho.

## Results

### Experiment 1: Acute Characterization

Twenty mice were operated. None of the animals displayed hemorrhagic signs on early MRI or on histology. MRA revealed no or incomplete occlusion in 2 animals, with visible signal in distal MCA branches. Minor perfusion defect (<10% of ipsilateral hemisphere) was present on MPC maps in 3 animals, including the 2 above-cited mice. These 3 animals showed no ADC-abnormal area and no histological infarct, and were excluded from subsequent volumetric analysis.

**Figure 1:**
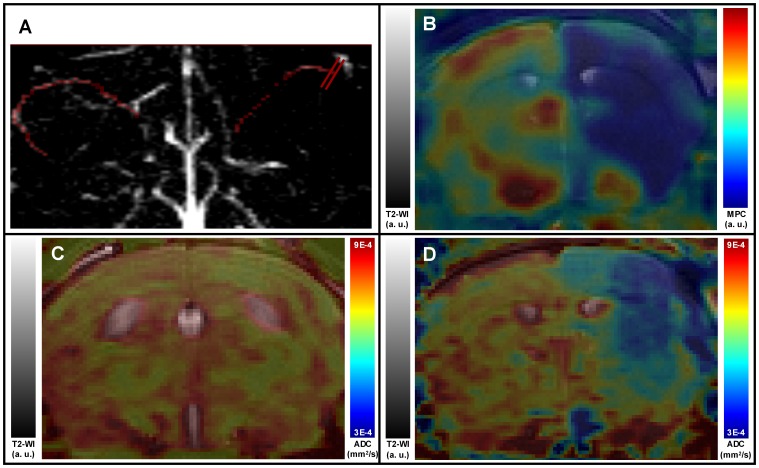
Acute multi-slice, multiparametric MRI of thromboembolic stroke in mice. (**A**) MR Angiography (maximum intensity projection). MCA signals were manually overlaid in red for clarity. Note the distally interrupted flow in the right MCA (bars). (**B**) Perfusion (MPC) map overlaid on corresponding T2-WI. Note the large perfusion defect in the right MCA territory (arrow) (**C**, **D**) Diffusion (ADC) maps overlaid on corresponding T2-WI. Note the cortico-striatal decrease in **D** (same animal as **B**), in contrast with **C**.

**Figure 2:**
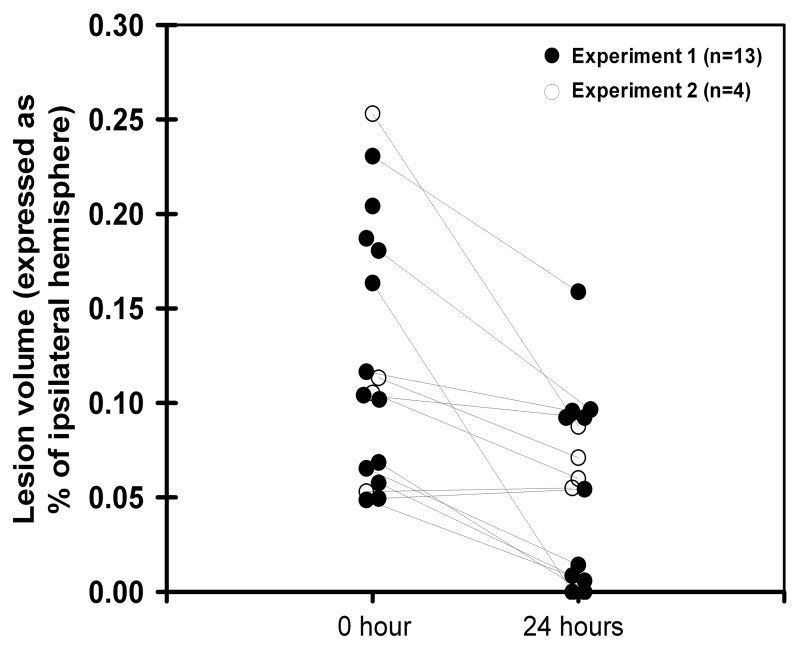
Pooled data from Experiment 1 (n = 13, full circles) and Experiment 2 (n = 4, open circles). Evolution of lesion volume (expressed as % of ipsilateral hemisphere) between 0 hour (ADC map) and 24 hours (histology in Experiment 1; T2-WI in Experiment 2). Only animals displaying a significant lesion beyond surgical site on ADC (i.e., lesion >5% of ipsilateral hemisphere) were considered. Two animals from Experiment 1 died shortly after MRI completion (no histological staining).

The remaining 17 mice demonstrated complete MCA occlusion (Fig. 1A), and extensive cortico-striatal hypoperfusion on MPC maps (Fig. 1B). Well-defined focal ADC lesions were observed in the cortex and the dorsal part of the striatum (Fig. 1D) in 13 animals, while ADC lesions were virtually absent or restricted to the surgical site (<5% of ipsilateral hemisphere, Fig. 1C) in 4 animals. Figure 2 (full circles) shows the evolution of ischemic infarct between 0 hour (ADC map) and 24 hours (histology) for the 13 mice with significant initial lesion (>5% of ipsilateral hemisphere): 2 animals died within 2 hours of ischemia, 6 animals showed cortical ischemic infarct after 24 hours, and 5 animals showed virtually no lesion.

The 15 animals that completed the protocol can therefore be classified among 3 groups depending on the presence of a significant ischemic area at H0 and H24:

- 6 animals had both an ADC lesion at H0 and a histological lesion at H24 (group A);
- 5 animals had an ADC lesion at H0 without a histological lesion à H24 (group B);
- 4 animals had no ADC lesion at H0 and no histological lesion at H24 (group C).

**Figure 3:**
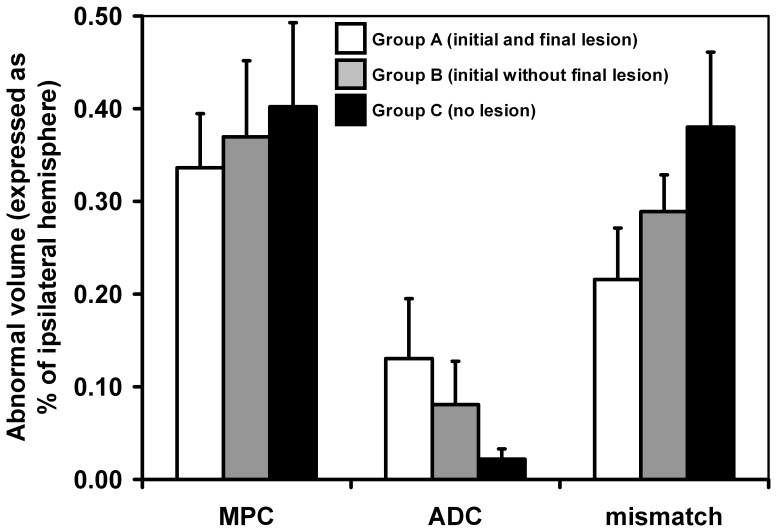
Quantification (mean±standard deviation) of initial MRI defects in Experiment 1 (n = 15): diffusion (ADC map), perfusion (MPC map) and perfusion-diffusion mismatch volumes, expressed as % of ipsilateral hemisphere. Mice were grouped according to presence/absence of ischemic lesion on baseline DWI and final histology. Group A (white bars, n = 6) = initial and final lesion; group B (gray bars, n = 5) = initial without final lesion; group C (black bars, n = 4) = neither initial nor final lesion (i.e., lesion <5% of ipsilateral hemisphere, confined to the surgical site).

Figure 3 summarizes initial MRI parameters in these groups. Regardless of initial or final ischemic area, the 3 groups shared similar MPC lesion volumes (P = 0.49). Of those animals displaying initial infarct on ADC, group B tended to have lower ADC lesion volumes and higher mismatch volumes than group A, but these differences did not reach significance (P = 0.18 and P = 0.06 respectively).

### Experiment 2: Extended Follow-up

**Figure 4:**
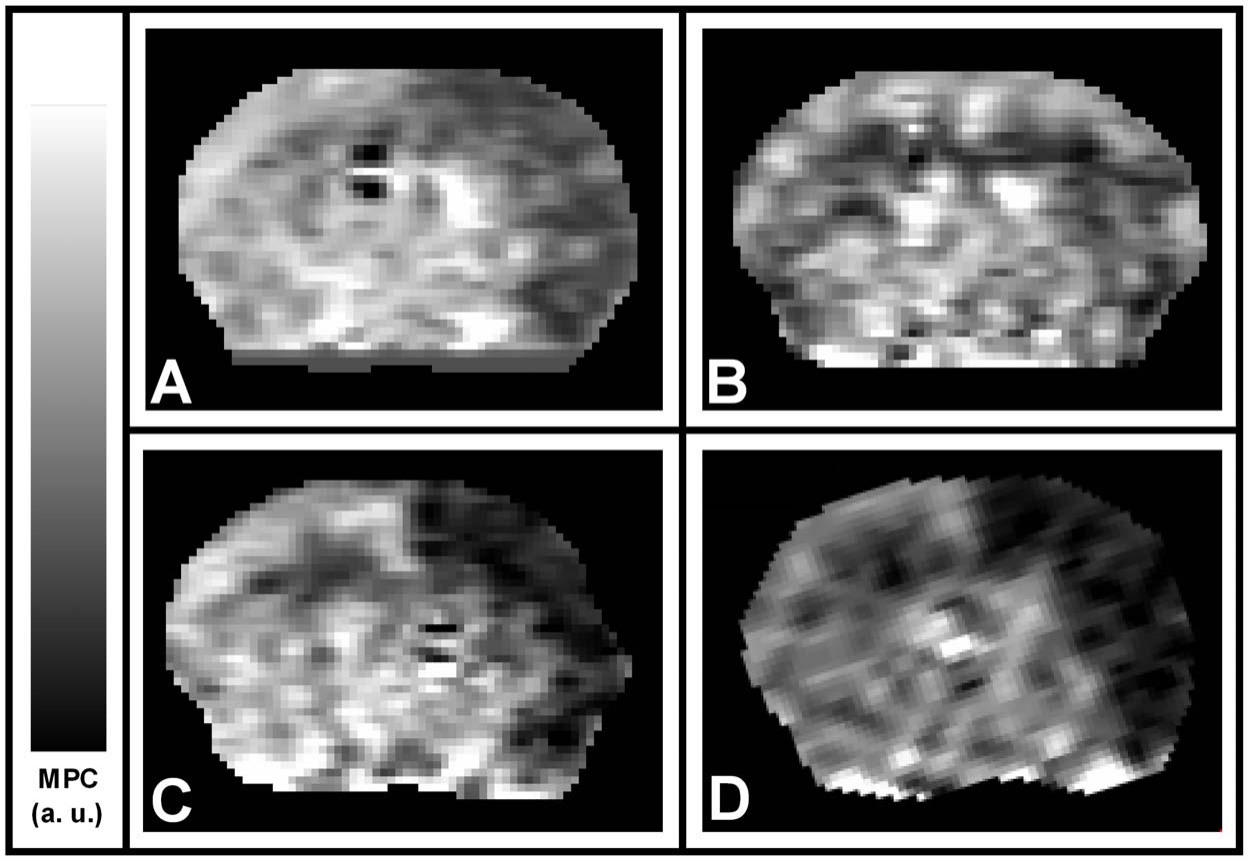
Single-slice perfusion (PASL) follow-up between 0 and 3 hours of thromboembolic stroke in mice. (**A**) CBF at 0 hour and (**B**) CBF after 3 hours in an animal with spontaneous reperfusion. (**C**) CBF at 0 hour and (**D**) CBF after 3 hours in an animal without reperfusion.

**Figure 5:**
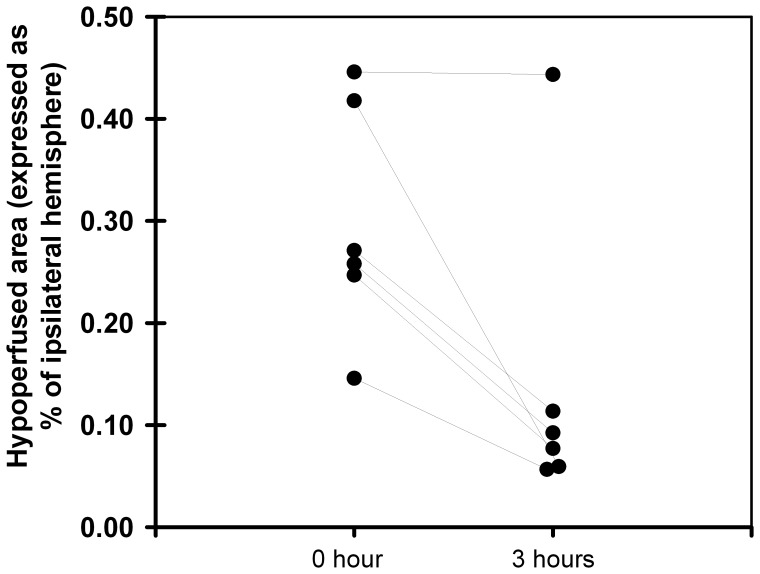
Evolution of hypoperfusion areas (expressed as % of ipsilateral hemisphere) between 0 and 3 hours in Experiment 2 (n = 6). CBF maps were thresholded at the 10^th^ percentile value of perfusion in the contralateral hemisphere.

Six mice were operated. At 0 hour, all exhibited a significant perfusion defect visualized on CBF maps (Fig. 4A, 4C and Fig. 5). Four animals exhibited an ADC lesion at 0 hour. In 3 out of 4 cases, this lesion strongly regressed after 3 hours (mean±SD = −83±23% of H0 area), but extended between 3 and 24 hours (mean±SD = −35±25% of H0 area). At 24 hours, T2-WI highlighted an intra-cerebral hemorrhagic area around the surgical site in 1 animal. Two animals had no ischemic lesions at any time point. Hence, in accordance with the results of Experiment 1, T2-WI infarction volumes after 24 hours were smaller than initial ADC lesion volumes (Figure 2, open circles), with the notable exception of the case of hemorrhagic transformation.

The evolution of the CBF defect during the first 3 hours following stroke onset is represented in Figure 5. Five animals showed spontaneous decrease in hypoperfusion area during the 3 hours (mean±SD = −68±11% of initial hypoperfused area), as illustrated in Fig. 4A–B. Only one animal exhibited sustained hypoperfusion (−1%, Fig. 4C–D) during the 3 hours. This evolution was not correlated with the development of infarction over time (Spearman correlation coefficients: between 0 and 3 hours, P = 0.20; between 0 and 24 hours, P = 0.80).

## Discussion

We report the first MRI characterization of a mouse model of *in situ* MCA occlusion [1]. MRA and PWI confirmed successful clot formation in 85% of animals, a rate similar to previous reports [2]. No intra-cranial hemorrhage was detected on MRI or histology, except in 1 animal. Likewise, previous studies reported no [1] or only mild [2] hemorrhage in untreated animals.

The first experiment, with single acute MRI examination, identified 3 groups of animals (Fig. 3): one with the expected early MRI and late histological lesion (group A), one with an MRI lesion without histological lesion (group B), and one without significant MRI or histological lesion (group C). In group C, no ischemic lesion developed despite a hypoperfusion volume similar to the other groups. One possible interpretation is that hypoperfusion in these animals may lie just below the penumbra threshold, preventing ADC lesion development. This hypothesis is difficult to confirm in rodents, as it would require an arterial input function for absolute quantification of perfusion. Even in patients, there is no widely accepted perfusion-derived threshold identifying penumbral tissue [4]. Similarly, the differential outcome between groups A and B could not be explained by initial MRI parameters (diffusion, hypoperfusion and mismatch volumes), despite a tendency for smaller ADC lesion volumes and higher mismatch volumes (Fig. 3). Spontaneous reperfusion occurring after MRI follow-up (>60 min) in group B is the likely explanation.

To confirm this hypothesis, a second experiment was conducted, with a sequential MRI protocol involving PASL-PWI and DWI repeated at 0 and 3 hours. One third of the animals did not develop a lesion, a proportion in the same range as in the first experiment. Drastic decrease in hypoperfusion volume (−68±11% of initial hypoperfusion) occurred during the first 3 hours following stroke onset in 80% of animals. This was accompanied by a transient regression of the ADC lesion. Spontaneous reperfusion thus contributed to the variability of ischemic lesion size in this model. Ischemia induction was performed according to the original description by Orset et al. [1], by injection of 0.75 U murine α-thrombin. Future studies could include higher doses of thrombin so as to induce more stable occlusion and, hopefully, a more reproducible infarct. Interestingly, implementation of the thrombin injection technique in macaques also resulted in frequently delayed reperfusion, despite a higher dose (800 U for 10 kg) [15].

Initial angiography was performed to determine surgical success. The angiographic signal was disturbed by extra-cranial micro-hemorrhages at the surgical site; however detection of unsuccessful occlusion relies on the visualization of small distal MCA branches, and is therefore very sensitive to image quality. Consequently, angiography proved to be of limited interest for identifying unsuccessful occlusion in this model. In contrast, PWI highlighted major and reproducible hemodynamic alterations in MCA territory which objectified successful occlusion (Fig. 1B and Fig. 3).

In conclusion, our main finding was a significant rate of spontaneous reperfusion in the first hours after the formation of a stable clot and subsequent MCA occlusion. This suggests, as in human stroke, differential evolution of the clot. This model appears invaluable for the evaluation of alternative thrombolytic therapies or neuroprotective drugs, provided that extended monitoring of reperfusion is performed to minimize variability.
